# Overcoming the Contact Problem in Quantitative Attenuated Total Reflection Spectroscopy Analysis of Flat Samples

**DOI:** 10.1177/00037028231199115

**Published:** 2023-09-12

**Authors:** Luis G. Vieira

**Affiliations:** 1 Centro de Física das Universidades do Minho e do Porto (CF-UM-UP), Laboratório de Física para Materiais e Tecnologias Emergentes (LaPMET) and Departamento de Física, 56059 Universidade do Minho, Braga, Portugal

**Keywords:** Infrared, IR, attenuated total reflection, ATR, contact problem, spectrum modeling, optical functions, polystyrene

## Abstract

A method for measuring the optical functions of a flat sample made of homogeneous and isotropic material, using attenuated total reflection spectroscopy when there is poor contact between the sample and the internal reflection element is presented. The approach consists in treating the spacing between the internal reflection element and the sample as an adjustable parameter, along with the dispersion model parameters, in the simultaneous fitting of *s*- and *p*-polarized spectra obtained when the gap distance is unknown. The method is tested with both synthetic and experimental (polystyrene) spectra. The results demonstrate the method's ability to accurately determine the optical functions even in the presence of a contact problem.

## Graphical abstract

**Graphical Abstract:**
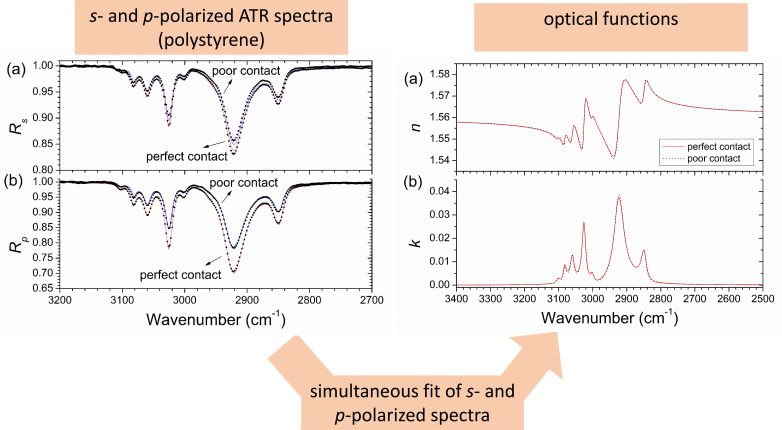
This is a visual representation of the abstract.

## Introduction

Attenuated total reflection (ATR) spectroscopy has become the preferred technique for studying the infrared (IR) absorption properties of materials due to its ability to analyze samples without requiring extensive preparation. The method has found widespread use in various fields, including biology, medicine, forensics, analytical and organic chemistry, and material science. Detailed explanations of this technique can be found in Harrick^1^ and Milosevic.^2^

Briefly, ATR utilizes the phenomenon of internal reflection that occurs when light passes from a higher refractive index medium to a lower one. The technique involves placing a sample in close contact with a transparent and high refractive index material, known as the internal reflection element (IRE), typically made of diamond or germanium. The absorption of the sample is then detected through the attenuation of total reflection manifested in the reflectance as dips below one in absorption regions. The most direct use of the technique consists of the location of the absorption bands of the studied material or a qualitative eye inspection of the intensity of the bands. For more quantitative analysis, it is possible to determine the optical functions of the materials by simulating the spectrum using Fresnel's equations and an appropriate dispersion model.^3^ However, if perfect contact between the sample and IRE is not ensured the results are not accurate.

The problem of achieving good contact between the sample and the IRE, commonly known as the contact problem, has been a well-known challenge since the development of the ATR technique.^4^ Over the years, efforts have been made to enhance the equipment's capability to achieve better contact and identify situations where contact is poor.^5–7^ While liquid samples typically provide good optical contact with the IRE, soft and rubbery solid samples may require pressure to achieve good contact. Hard and rigid solid samples, on the other hand, do not always make good contact with the IRE, especially if the IRE in use cannot support high pressures, and an unknown gap between the two media may remain. The contact problem also arises in situations where the sample is fragile, and there is a need to avoid breaking it under the contact force applied during the ATR technique. If the spacing resulting from poor contact between the sample and the IRE is not considered, significant errors may occur in determining the optical functions.

This work introduces a novel approach to address the contact problem in ATR measurements through numerical simulation of the spectra in the case of isotropic and homogeneous samples. The proposed method enables the determination of both the gap between the sample and IRE, as well as the optical functions of the material.

## Materials and Methods

**Figure 1.**
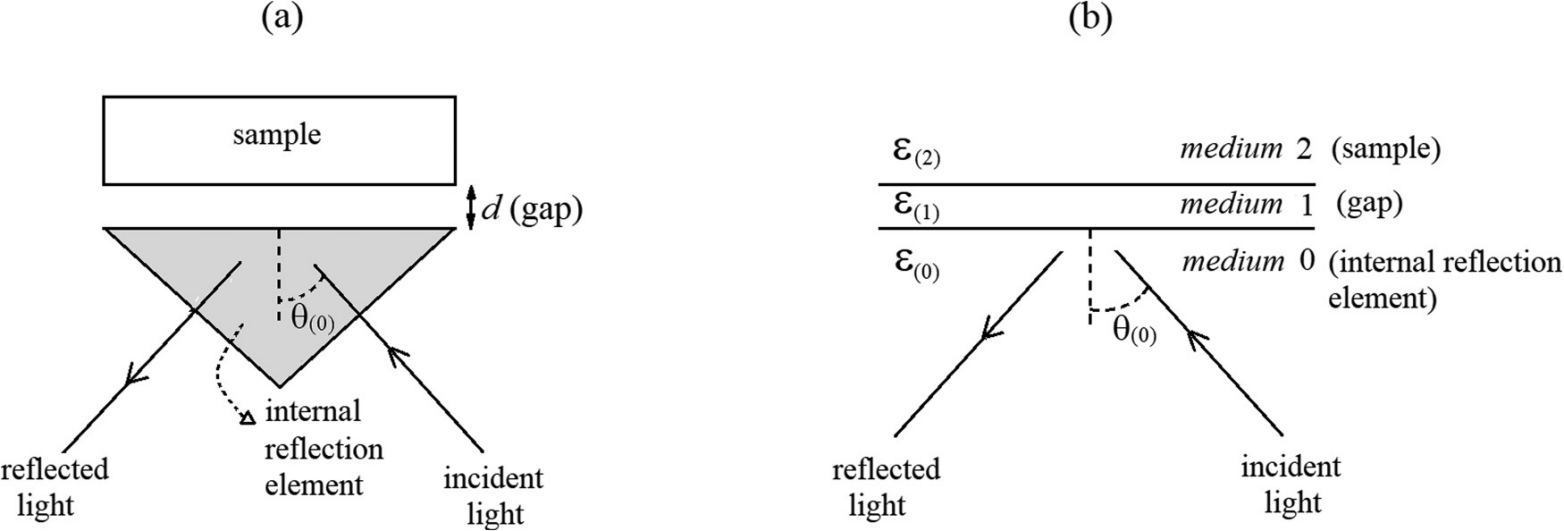
(a) Configuration of the single reflection ATR system: the dielectric constant sample ε_(2)_ is placed at a distance *d* (gap) from the top surface of an IRE of dielectric constant ε_(0)_; the gap is usually air or vacuum (dielectric constant ε_(2)_ = 1). (b) Equivalent optical configuration of the ATR system with a uniform gap. When the sample is in perfect contact with the IRE the gap is absent (*d *= 0).

The IR ATR spectra of polystyrene (a slab of dimensions approximately 1 cm × 1 cm × 0.2 cm) were measured with a Golden Gate single-reflection ATR system (Specac) mounted in a Fourier transform IR spectrometer (Bruker IFS 66 V). In this standard ATR system light is passed through the IRE, that is, a prismatic diamond crystal, and reaches the interface with a top second medium at a nominal angle of 45° (the scheme of the optical configuration of the system is depicted in Figure 1a. It is assumed that the diamond crystal is transparent and presents a nearly constant refractive index (2.40) in the scanned spectral regions (2500–3400 cm^–1^). As is typical in commercial ATR accessories, the system utilized in this study is equipped with a pressure control mechanism that can apply a certain maximum pressure to achieve perfect optical contact between the IRE and a flat sample. The same mechanism was utilized to adjust the gap (*d*) between the diamond crystal and the sample in this study, even though the value of *d* is unknown.

The measurements were performed with a globar source, a potassium bromide (KBr) beamsplitter, a KRS-5 polarizer, and a deuterated triglycine sulfate detector with a KBr window. The resolution was better than 4 cm^–1^. The background spectral intensity was measured prior to the sample placement when the conditions of total internal reflection are satisfied. When the absorbing sample was brought into contact or close to the surface of the IRE, the reflectance of the corresponding spectral intensity was recorded. The ATR spectrum is the ratio of the later and the former spectral intensities. Finally, since the reflectance was slightly below one at the transparent highest wavenumber region, where total reflection should be observed, the spectra were corrected by multiplying by factors <1.0165 to bring the reflectivity close to one at a high wavenumber.

Considering the experimental setup (Figure 1a) and the measurement procedure, the ATR can be modeled by considering the equivalent optical system shown in Figure 1b. This equivalent arrangement consists of three isotropic and homogeneous media, with parallel interfaces: semi-infinite medium 0 (diamond, which constitutes the IRE), medium 1 (vacuum gap), and semi-infinite medium 2 (sample). The procedure here adopted to model the poor contact ATR spectra, based on Fresnel equations and dielectric function models, is similar to the method for perfect contact conditions discussed in detail by Vieira^3^), except that now an additional layer is introduced to describe the effect of a gap between the IRE and the sample.

Let ε_(*j*)_ denote the complex dielectric function of medium *j* (*j* = 0, 1, 2); φ_(*j–*1)_ and φ_(*j*)_ denote the angles of incidence and refraction, respectively, when the light comes from medium *j* *–* 1 to medium *j* (*j* = 1, 2). The complex refractive index of medium *j* is

(1) $${\tilde{n}}_{\left(j\right)}=n_{\left(j\right)}+ik_{\left(j\right)}$$

and is related to the complex dielectric function

(2) $$\varepsilon_{\left(j\right)}=\;{\varepsilon^{\prime }}_{\left(j\right)}\;+\;i{\varepsilon^{\prime \prime }}_{\left(j\right)}$$

by

(3) $${\tilde{n}}_{\left(j\right)}=\sqrt{\varepsilon_{\left(j\right)}}$$

The complex reflection coefficients of this system (a layer between two semi-infinite media), for *s*- and *p*-polarized light, *r_s_* and *r_p_*, respectively, can be calculated from the boundary conditions for electric and magnetic fields. Denoting by *r_k_* the reflection coefficient for *k* (*k* = *s*, *p*) polarized light, the following well-known expression is obtained^8^:

(4) $$r_{k}=\frac{r_{k(1)}+r_{k(2)}e^{2i\delta }}{1+r_{k(1)}r_{k(2)}e^{2i\delta }}$$

where

(5) $$\delta =2\pi \tilde{\nu }{\tilde{n}}_{\left(1\right)}d\cos\;\varphi_{\left(1\right)}$$

The $\tilde{\nu }$ and *d* are the wavenumber (the reciprocal of wavelength in vacuum) and the thickness of the layer (medium 1), respectively. The reflection coefficients *r_k_*_(*j*)_ (*k* = *s*, *p*; *j* = 1, 2) are given by the Fresnel equations (see, e.g., Born and Wolf^9^)

(6) $$r_{s(j)}=\frac{{\tilde{n}}_{\left(\;j-1\right)}\cos\;\varphi_{\left(\;j-1\right)}-{\tilde{n}}_{\left(j\right)}\cos\;\varphi_{\left(j\right)}}{{\tilde{n}}_{\left(\;j-1\right)}\cos\;\varphi_{\left(\;j-1\right)}+{\tilde{n}}_{\left(j\right)}\cos\;\varphi_{\left(j\right)}}$$

(7) $$r_{\;p(j)}=\frac{{\tilde{n}}_{\left(\;j-1\right)}\cos\;\varphi_{\left(j\right)}-{\tilde{n}}_{\left(j\right)}\cos\;\varphi_{\left(\;j-1\right)}}{{\tilde{n}}_{\left(\;j-1\right)}\cos\;\varphi_{\left(j\right)}+{\tilde{n}}_{\left(j\right)}\cos\;\varphi_{\left(\;j-1\right)}}$$

The relation between the refraction angle and the incidence angle is given by Snell's law:

(8) $${\tilde{n}}_{\left(\;j-1\right)}\sin\;\varphi_{\left(\;j-1\right)}={\tilde{n}}_{\left(j\right)}\sin\;\varphi_{\left(j\right)}$$

Notice that media 0 and 1 are transparent so that ε_(0)_, ε_(1)_, φ_(0),_ and φ_(1)_ are real.

The reflectance for *s*- and *p*-polarization is given by

(9) $$R_{s}=|r_{s}{|}^{2}$$

and

(10) $$R_{p}=|r_{p}{|}^{2}$$

respectively. The nonpolarized reflectance, *R*, is obtained by considering 50% *s-*polarization–50% *p*-polarization of the incident beam:

(11) $$R=\frac{1}{2}(R_{s}+R_{p})$$

To describe a perfect contact configuration (no gap) Eqs. 4 and 5 are omitted and Eqs. 6, 7, and 8 are taken between the contiguous semi-infinite media that constitute the IRE and the sample.

The various spectra *R_s_*, *R_p_*, and *R* can be modeled by using Eqs. 1 to 11 and an appropriate model for the dielectric function of the media. In the present work, the permittivity of media 0 (diamond) and 1 (vacuum) are independent of the wavenumber (ε_(0)_ = (2.40)^2^ = 5.76, ε_(1)_ = 1), whereas the permittivity of medium 2 (sample) is taken to depend on wavenumber ( $\tilde{\nu }$ ) according to the factorized form of the dielectric function:^10^

(12) $$\varepsilon (\tilde{\nu })=\varepsilon_{\infty }\overset{n}{\underset{\;j=1}{\prod }}\frac{\Omega_{Z_{j}}^{2}-{\tilde{\nu }}^{2}+i\tilde{\nu }\gamma_{Z_{j}}}{\Omega_{P_{j}}^{2}-{\tilde{\nu }}^{2}+i\tilde{\nu }\gamma_{P_{j}}}$$

where Ω*_Zj_* and Ω*_Pj_* are the zeroes and poles of the dielectric function, respectively, γ*_Zj_* and γ*_Zj_* are damping coefficients, and ε_∞_ is the high-wavenumber dielectric constant (commonly at optical frequencies). This dispersion model is highly versatile, capable of representing a wide range of dielectric responses. It is frequently utilized to characterize the optical properties of crystals and to derive parameters for optical modes; in such cases, Ω*_Pj_* and Ω*_Zj_* are typically assigned to the frequencies of transverse and longitudinal optical modes, respectively.

In this study, the ATR spectra were modeled using a nonlinear regression fitting algorithm implemented in the open-source software GNU Octave,^11^ specifically using the Levenberg–Marquardt algorithm^12^ through the Octave function “leasqr”. The uncertainty of the fitted parameters is the standard deviation taken as the square root of the diagonal elements of the covariance matrix.

## Exploiting the Effect of Spacing in ATR Measurements

**Figure 2.**
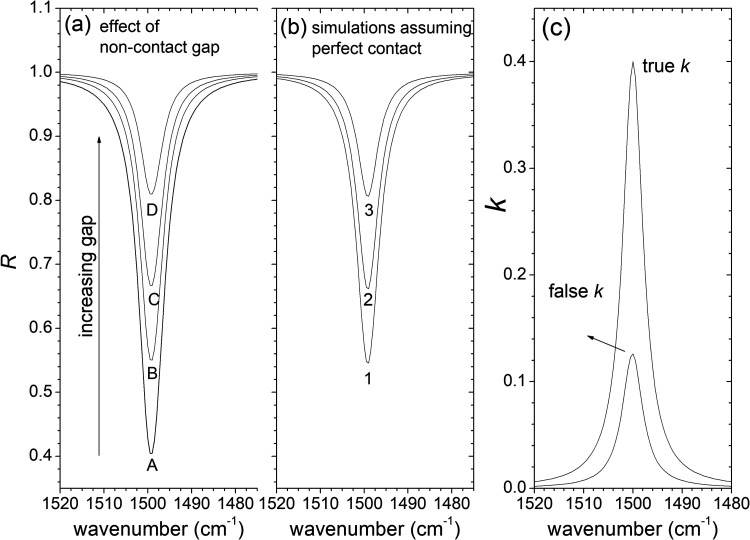
(a) Attenuated total reflectance (ATR) of a model one mode system with the following parameters of Eq. 12: Ω*_P_* = 1500 cm^–1^, Ω*_Z_* = 1500.4 cm^–1^, γ*_P_* = γ*_Z_* = 5 cm^–1^ and ε_∞_ = 2.5, for several thicknesses (*d*) of noncontact gap (*d* = 0, 100, 200, and 400 nm, corresponding to the spectra identified with *A*, *B*, *C*, and *D*, respectively. (b) Spectra *1*, *2*, and *3* are simulations of spectra *B*, *C*, and *D* of Figure 2a, respectively, assuming perfect contact conditions (no gap) and different model parameters. (c) Imaginary part of the refractive index is used to calculate the ATR spectra in Figure 2a (true *k*) and spectrum *1* of Figure 2b (false *k*).

The first step in this study is to analyze how the vacuum gap between the IRE and the sample affects the outcome spectrum and the optical functions evaluated if the gap, *d*, is not properly considered. For this purpose, we use calculated (synthetic) ATR spectra considering a simple model medium with one mode, constructed using Eqs. 1 to 12. Figure 2a illustrates the effect of varying *d* on nonpolarized spectra. The spectra A, B, C, and D were calculated using the same model parameters (Ω*_P_* = 1500 cm^–1^, Ω*_Z_* = 1500.4 cm^–1^, γ*_Z _* = γ*_P _* = 5 cm^–1^, ε_∞_ = 2.5 in Eq. 12) for all situations and varying *d* from zero (spectrum A with no spacing) to 400 nm. Spectra B (*d *= 100 nm), C (*d *= 200 nm), and D (*d* = 400 nm) were then fitted ignoring the gap (spectra 1, 2, and 3 of Figure 2b are fits of spectra B, C, and D, respectively). It turns out that it is possible to reproduce quite well the original spectra obtained with noncontact gaps by assuming perfect contact as long as the model parameters are adjusted: the effect on the spectrum of varying *d* is similar to the effect of changing appropriately the parameters of the dispersion model. In this particular example, the discrepancies between the values of the reflectance obtained by the two ways are <1%. This shows that when analyzing real experimental spectra (with noise), achieving excellent contact between the sample and the IRE (or knowing the gap a priori) is necessary for rigorously extracting the optical functions from nonpolarized measurements. Neglecting the spacing can lead to significant distortions in determining the optical functions, as shown in Figure 2c. Here the imaginary part of the refractive index (*k*) obtained directly from the model parameters used to construct the spectra A, B, C, and D in Figure 2a (true *k*) is compared with *k* calculated from the fitted spectrum 1 in Figure 2b (false *k*). It is clear that, despite the small value of the gap (100 nm), disregarding it in the fit can introduce important deviations in the determination of the optical functions.

**Figure 3.**
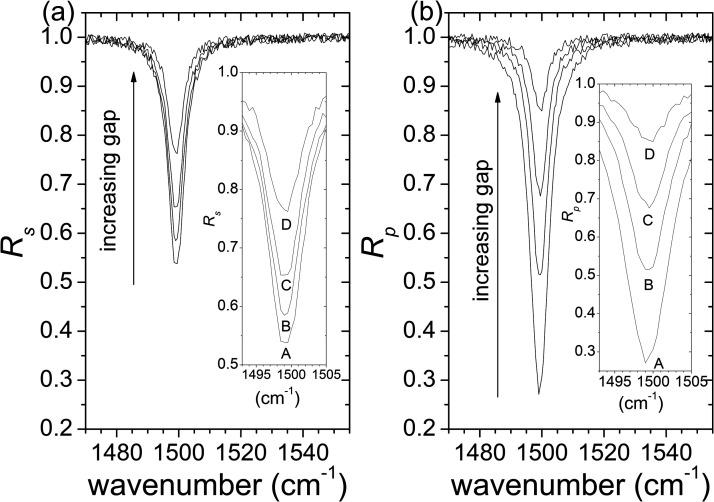
Calculated ATR spectra of one mode system for *s*-polarization (a) and *p*-polarization (b) as a function of the thickness, *d*, of a noncontact gap; the following parameters of Eq. 12 were used: Ω*_P_* = 1500 cm^–1^, Ω*_Z_* = 1500.4 cm^–1^, γ*_P_* = γ*_Z_* = 5 cm^–1^, ε_∞_ = 2.5. Generated noise with an amplitude <0.01 was added to all spectra. The insets show the detail of spectra around the absorption band; the gap distances *d* = 0, 100, 200, and 400 nm, correspond to the spectra identified with *A*, *B*, *C*, and *D*, respectively.

The main idea of this study is to address the contact problem in ATR spectroscopy by using the gap as a fitting parameter when modeling the spectrum. The previous analysis has shown that determining the dielectric function unambiguously from a nonpolarized spectrum with poor contact is not feasible, because equal good fits can be achieved with different gaps. However, incorporating additional spectroscopic information from *s*- and *p*-polarized spectra can help overcome this problem. Figure 3 displays the dependence of synthetic *s*- and *p*-polarized spectra on the gap distance *d* (the same parameters as before (Figure 2) were used to calculate the spectra, but now the *s*- and *p*-polarized spectra are considered separately). To simulate realistic data, a noise with an amplitude of <0.01 was added. As shown in Figure 3, the spectral changes in *s*- and *p*-polarized spectra are distinct when the gap distance is varied. The proposed method is to treat the gap distance as an adjustable parameter, along with the dispersion model parameters, in the simultaneous fitting of *s*- and *p*-polarized spectra obtained when the gap distance is unknown.

**Figure 4.**
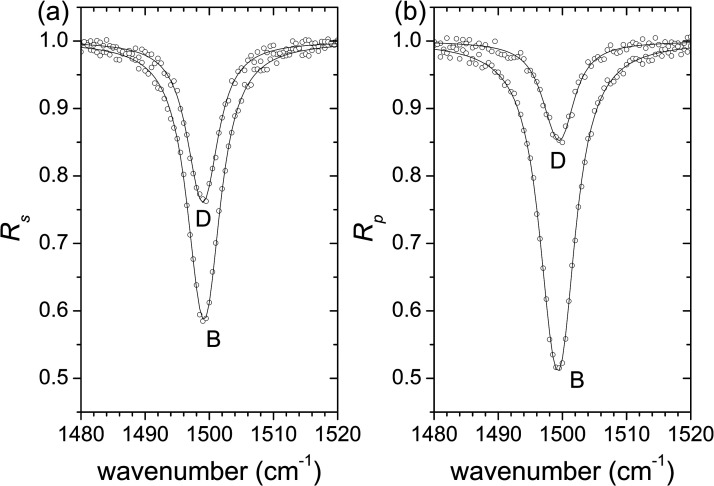
Synthetic spectra for (a) *s*- and (b) *p*-polarized light (dots; spectra marked with *B* and *D* were constructed with vacuum gaps *d* = 100 and 400 nm, respectively) and fitted spectra obtained from the simultaneous fit of the two spectra (lines).

In order to test the method, the simultaneous fit of pairs of *s*- and *p*-polarized spectra were performed for the cases of spectra A, B, C, and D. It was found that departing from different initial sets of parameters led to unique final fitted sets of parameters which are in excellent agreement with those used to produce the synthetic spectra (expected parameters). The fitted and expected parameters for the cases of spectra A, B, C, and D are shown in Table I. The uncertainties shown are the standard deviations. It is observed that the errors in the parameters (difference between expected and fitted values) are less than one standard deviation for cases A, B, and C, and are less than two standard deviations for case D. Figure 4 presents, by way of example, *s*- and *p*-polarized spectra constructed with *d* = 100 and 400 nm (spectra B and D, respectively, in Figure 3) and the corresponding fits.

**Table I.** Fitted and expected parameters obtained from the simultaneous fit of *s*- and *p*-polarized synthetic spectra A, B, C, and D of Figure 3. The uncertainties of the fitted parameters are the standard deviations.

Parameters		Ω*_P_* (cm^–1^)	Ω*_Z_* (cm^–1^)	γ*_P _* = γ*_Z_* (cm^–1^)	*d* (nm)	Expected *d* (nm)
expected		1500.00	1500.40	5.00		
Fitted	Spectra A	1500.00 ± 0.01	1500.40 ± 0.01	5.05 ± 0.03	–1 ± 1	0
	Spectra B	1500.01 ± 0.02	1500.42 ± 0.02	4.99 ± 0.03	101 ± 2	100
	Spectra C	1499.99 ± 0.02	1500.39 ± 0.02	4.98 ± 0.04	200 ± 3	200
	Spectra D	1499.92 ± 0.04	1500.31 ± 0.04	5.00 ± 0.07	386 ± 9	400

## Experimental Test of the Method

The experimental test of the method previously described is performed by using spectra of a solid polystyrene slab taken in the spectral region 2500–3400 cm^–1^. In this region, polystyrene exhibits a set of absorption bands ascribed to C–H stretching vibrations which are well separated from other absorption bands occurring at lower wavenumber. To begin with, an accurate determination of the dielectric function is performed from perfect contact (no spacing) spectra by simultaneously modeling the *s*- and *p*-polarized spectra. Then, polarized spectra taken at lower contact forces are analyzed. The optical functions obtained with noncontact ATR are compared with those obtained with perfect contact ATR spectra. The mechanical characteristics of the sample are quite adequate to test the method because solid polystyrene is flexible enough to guarantee excellent contact with the diamond IRE when maximum force is exerted by the pressure control mechanism of the equipment. On the other hand, the uniformity of the gap between the IRE and the sample, a requisite assumed by the model previously described, is not a priori ensured, but the flatness of the sample surface is a fundamental characteristic to materialize that condition.

**Figure 5.**
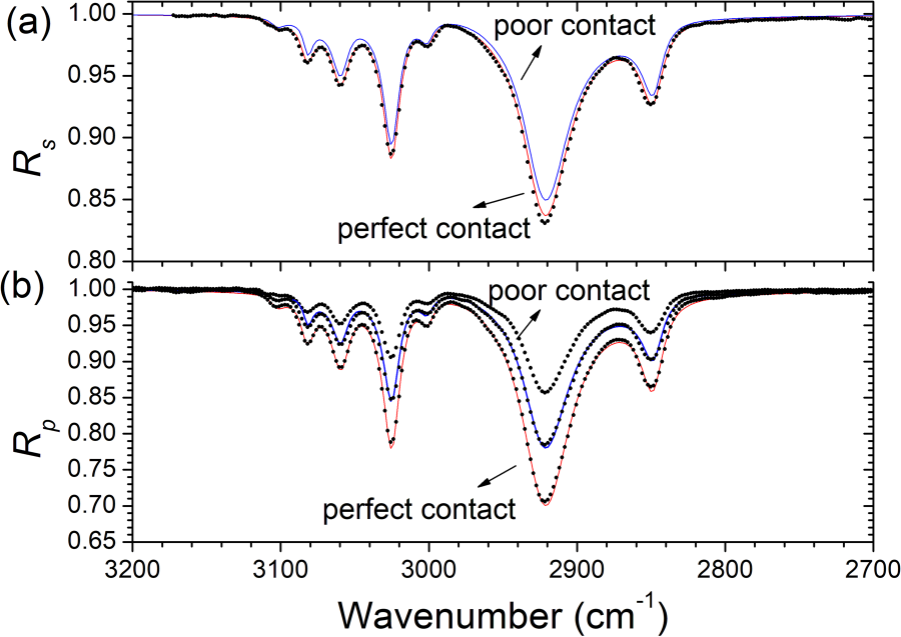
(a) Experimental (dots) and fitted (lines) ATR spectra of polystyrene for (a) *s*- and (b) *p*-polarizations acquired in perfect and poor contact conditions in the spectral range 2500–3400 cm^–1^.

Figure 5 shows the *s*- and *p*-polarized spectra of polystyrene taken at the maximum applied contact force (perfect contact) and at a lower contact force (poor contact). As expected the poor contact spectra exhibit less intense bands. The spectra were modeled using Eqs. 1 to 10 and 12 to extract the optical functions for both contact and noncontact spectra. In both cases, excellent fittings of the spectra were obtained, as also shown in Figure 5, by fitting the dispersion model parameters of Eq. 12. Notice that the parameter ε_∞_ (high-frequency dielectric constant) was not allowed to vary in the fitting procedure, because it is not possible to extract it from ATR measurements^3^; ε_∞_ was assigned a value of 2.434, the square of the refractive index of polystyrene at about 6000 cm^–1^ taken from Zhang et al.^13^ The gap *d* was taken as zero in the case of perfect contact conditions and as a fitting parameter in the case of noncontact spectra. In the latter case, the fitted value of the vacuum gap is *d* = 32 nm.

**Figure 6.**
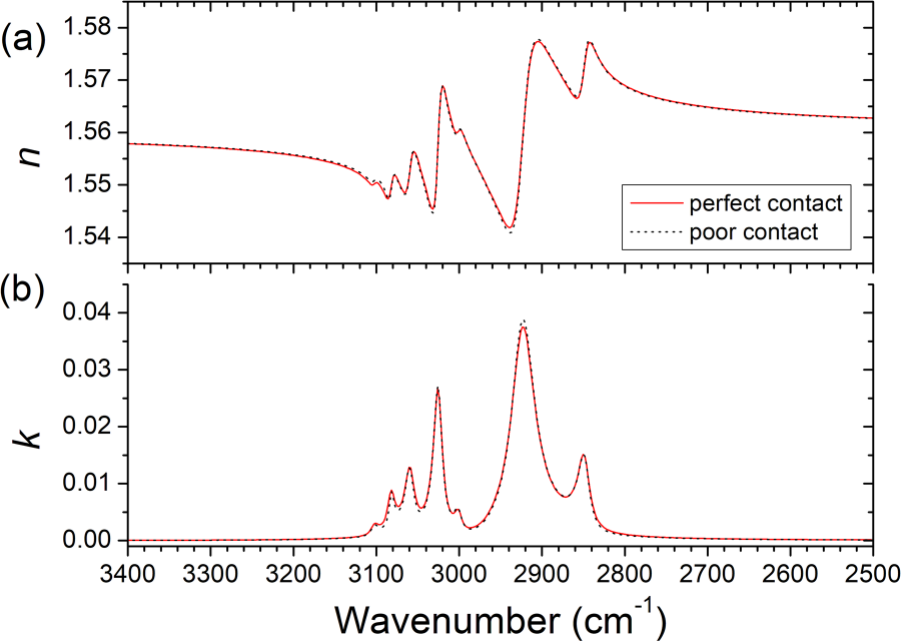
Real (*n*) and imaginary (*k*) parts of the refractive index of polystyrene obtained from simultaneous fitting of the *s*- and *p*-polarized ATR spectra of Figure 5 obtained in perfect contact (line) and poor contact (dots) conditions.

Figure 6. presents the real (*n*) and imaginary (*k*) parts of the refractive index obtained from the fits of the spectra. These results are consistent with those reported by Zhang et al.^13^ The main absorption bands are located at the wavenumbers 2850, 2923 3026, 3060, and 3081 cm^–1^ in agreement with the published results by Liang and Krimm.^14^

Most importantly, the optical functions obtained with perfect contact and poor contact measurements are almost undistinguishable, proving the effectiveness of the method. However, it must be noticed that experimental tests made with much lower contact forces (much higher gap) led to spectra that were not possible to fit. Probably this was due to the appearance of a nonuniform gap when the sample was more detached from the IRE. An experimental criterion to decide whether the conditions may be appropriate to apply the present method is that the intensity of *s*- and *p*-polarized spectra should be clearly different. Whenever the spectra for both polarizations become similar, probably, the sample is already very loose, and the conditions of a uniform gap are not guaranteed.

While ATR spectra of polystyrene in the spectral region 2500–3400 cm^–1^, with relatively strong reflection bands and a high signal-to-noise ratio (S/N), yield satisfactory results, analyzing poor-quality spectra with a low S/N can pose challenges regarding the accuracy of the determined optical functions. To address this issue, an improvement of the method can be used that involves obtaining *s*- and *p*-polarized spectra for two unknown gaps achieved by applying different contact forces on the sample, rather than just one unknown gap. The analysis can then be performed by simultaneously fitting four spectra (two *s*- and two *p*-polarized spectra corresponding to the two unknown spacing configurations). By doing so, more accurate optical functions of the material can be determined along with the values of the two unknown gaps.

## Conclusion

The effect of the spacing between the IRE and the sample in ATR measurements has been discussed. The results emphasize that neglecting the gap can lead to significant errors in determining the optical functions. To address this issue, a novel method was proposed for the noncontact ATR spectroscopy of flat samples made of homogeneous and isotropic material. The method treats the gap distance as an adjustable parameter in addition to the dispersion model parameters, in the simultaneous fitting of *s*- and *p*-polarized spectra.

Experimental tests were conducted on a solid polystyrene slab to validate the effectiveness of the proposed approach. The results show that the method accurately determines the optical functions from noncontact ATR spectra. The introduced method provides an effective solution for situations where perfect contact conditions are uncertain or unattainable. With this approach, spectroscopists can confidently analyze ATR spectra and extract optical functions, regardless of whether perfect contact was achieved during the experiment.
